# Experimental and numerical study on DCB specimens bonded with similar and dissimilar materials

**DOI:** 10.1016/j.heliyon.2022.e11037

**Published:** 2022-10-10

**Authors:** Mingze Ma, Zhiqiang Zhou, Wen Jiang, Weixing Yao, Piao Li

**Affiliations:** a State Key Laboratory of Mechanics and Control of Mechanical Structures, Nanjing University of Aeronautics and Astronautics, Nanjing 210016, China; b Key Laboratory of Fundamental Science for National Defense-Advanced Design Technology of Flight Vehicle, Nanjing University of Aeronautics and Astronautics, Nanjing 210016, China; c Aviation Key Laboratory of Science and Technology on Aero Electromechanical System Integration, Nanjing Engineering Institute of Aircraft Systems, AVIC, Nanjing 211106, China

**Keywords:** Bonded structure, Finite element method, Material randomness, Interface failure mode

## Abstract

In this paper, the influence of the bonding materials on the failure modes and the critical energy release rate (CERR) is studied through the double cantilever beam (DCB) test. The test results show that the failure mode and CERR of the bonded structure are closely related to the bonding materials, and three failure modes, i.e., the cohesive failure, the interface failure and the mixed-mode failure are identified on the bonding surface. The finite element method is used to simulate the interface debonding behavior of the DCB test specimens, and the influence of material randomness on the interface failure is introduced. A XFEM/CZM coupled approach is proposed to model the crack migration phenomena. The predicted results have a good agreement with the experimental results.

## 1 Introduction

Nowadays, a large number of composite materials have been used in engineering fields. This has also led to the widespread use of different bonding technologies in the adhesive connection of structures.

At present, many researchers have studied the bonding properties of composite materials. The delamination behavior of composite materials is influenced by the stacking sequence and fiber directions [1]. Kim et al. [2, 3, 4, 5] studied the effect of fiber direction and stacking sequence on the interface properties of carbon fibre reinforced plastics (CFRP) through experiments. Rehan [6] studied the effects of fiber orientation on mode I crack in carbon-epoxy laminates and found that different subsequent ply orientations lead to different crack resistance behavior. Gong [7, 8] experimentally investigated the mechanisms of delamination in multidirectional specimens and found that delamination migration would increase the fracture toughness. Brunner [9] performed test of symmetric and non-symmetric laminates and observed some fibre-bridging by fibre bundles. Pereira [10] and Zhao [11] found that the initial fracture toughness had no relevance to the ply angle. de Morais [12] and Laksimi [13] investigated delamination behavior of laminates with different layups and found that fracture toughness of 90° interfaces were higher than 0° specimens. In contrast, Ozdil [14] found that the initial fracture toughness decreased with the increasing of ply angle. Krueger [15] reviewed the history, approach and applications of VCCT, and discussed the problems associated with cracks propagating between different materials. Cricrì [16] and Perrella [17] proposed a novel identification method of CZM parameters and compared with approaches previous research. Rarani [18] studied the finite element model strategies of DCB test and discussed the advantages and limitations of VCCT, CZM and XFEM. Ricco [19, 20] considered the effect of fiber bridging behavior of delaminated laminates and numerically simulated the experiments.

The bonding between different materials such as metal and concrete is also common in engineering practice. Zhang [21] studied the debonding behavior between the FRP and concrete through a one-sided shear test, and used the DIC technology to obtain the full-field deformation. Nao [22] investigated the failure behavior of the similar and dissimilar material adhesive joints through DCB and TDCB(Tapered-DCB) tests. Pang [23] experimentally studied the debonding behavior of the CFRP-steel structure under quasi-static cyclic loading, and analyzed the interfacial damage and the threshold load. Hugo [24] proposed an analytical approach for prediction of debonding of steel-FRP interfaces. Yang [25] studied the fatigue performance of CFRP-steel adhesive joints under cyclic shear loading through experiments. Imanaka [26] studied the fatigue crack growth of CFRP-Al (Aluminium) DCB test specimens. Sellitto [27] numerically investigated the stringer termination delamination in tensile loaded hybrid metallic-CFRP stiffened aeronautical panel with VCCT.

In this paper, the debonding behavior of CFRP-CFRP, Al–Al and Al-CFRP specimens is studied by DCB test. The differences in loading curves, failure modes and CERRs of different specimens are compared. At the same time, the CZM, VCCT and XFEM modeling methods are adopted to simulate different debonding behavior. The randomness of material properties is introduced to account for the unstable propagation in the interface. A XFEM/CZM coupled approach is proposed to predict the crack migration in Al-CFRP specimen.

## 2 Experiment and results

### 2.1 Specimen

**Figure 1:**
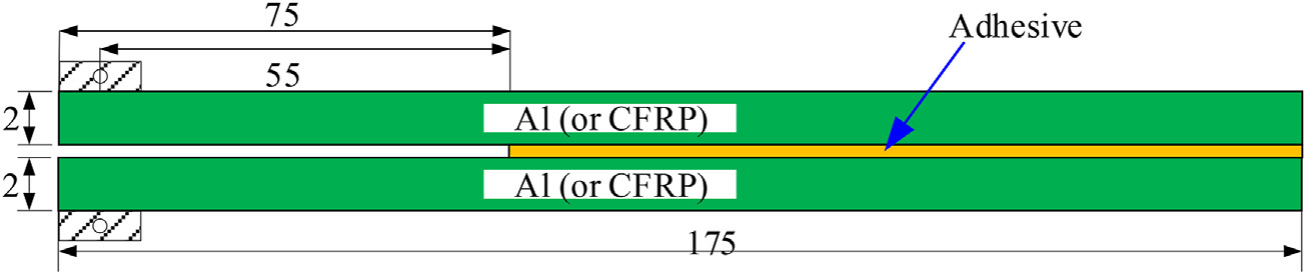
Sketch of DCB specimen.

The geometry of the DCB specimen is shown in Figure 1. The material of the cantilever beam includes the 7075 aluminum alloy and CFRP. Through the different combination of these two materials, there are three different types of specimens, i.e., the Al–Al, Al-CFRP, and CFRP–CFRP type and one specimen is tested for each type. The bonding surface was sanded rough to obtain better bonding performance. All the specimens are bonded with acrylic adhesive (Ergo® 1307) and cured for one week at room temperature with a cure pressure of 0.1 MPa.

The total length of the specimen is 175 mm, the width is 30 mm, and the length of the pre-crack is 75 mm. The distance from the loading point to the tip of the initial crack is 55 mm. The thicknesses of the aluminum plate and the composite laminate are both 2 mm. The elastic modulus of the aluminum is 72 GPa, and the Poisson’s ratio is 0.33. The composite laminate is T300/Epoxy, and the stacking sequence is [0,90]_8s_. The material properties of the CFRP unidirectional board are listed in Table 1.

**Table 1:** Material constants of unidirectional CFRP laminate [28].

*E*_1_ (GPa)	*E*_2_ (GPa)	*μ*_12_	*G*_12_ (GPa)
125	8.9	0.36	5.5

According to the classical laminate theory (CLT), the equivalent elastic modulus of the CFRP laminate is calculated to be 67.4GPa.

### 2.2 Experiment setup

**Figure 2:**
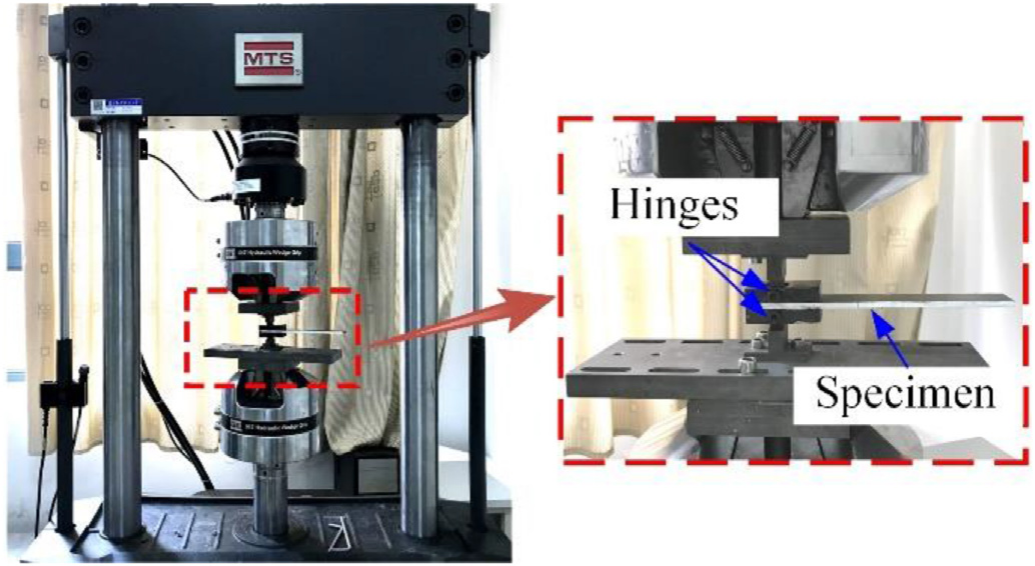
Experiment setup of DCB test.

As shown in Figure 2, The DCB test was carried out on a MTS 370.05 testing machine in the present study. The loading block on the specimen was hinged with the fixture on the test machine through bolts. At the same time, in order to facilitate the observation of crack propagation in the specimen, the observation side of the specimen was painted white, and the scale lines were marked with pencil. The testing machine was loaded at a rate of 0.5 mm/min until both parts of the test piece were completely separated, and the test data was collected at a rate of 5Hz.

### 2.3 Experiment results

**Figure 3:**
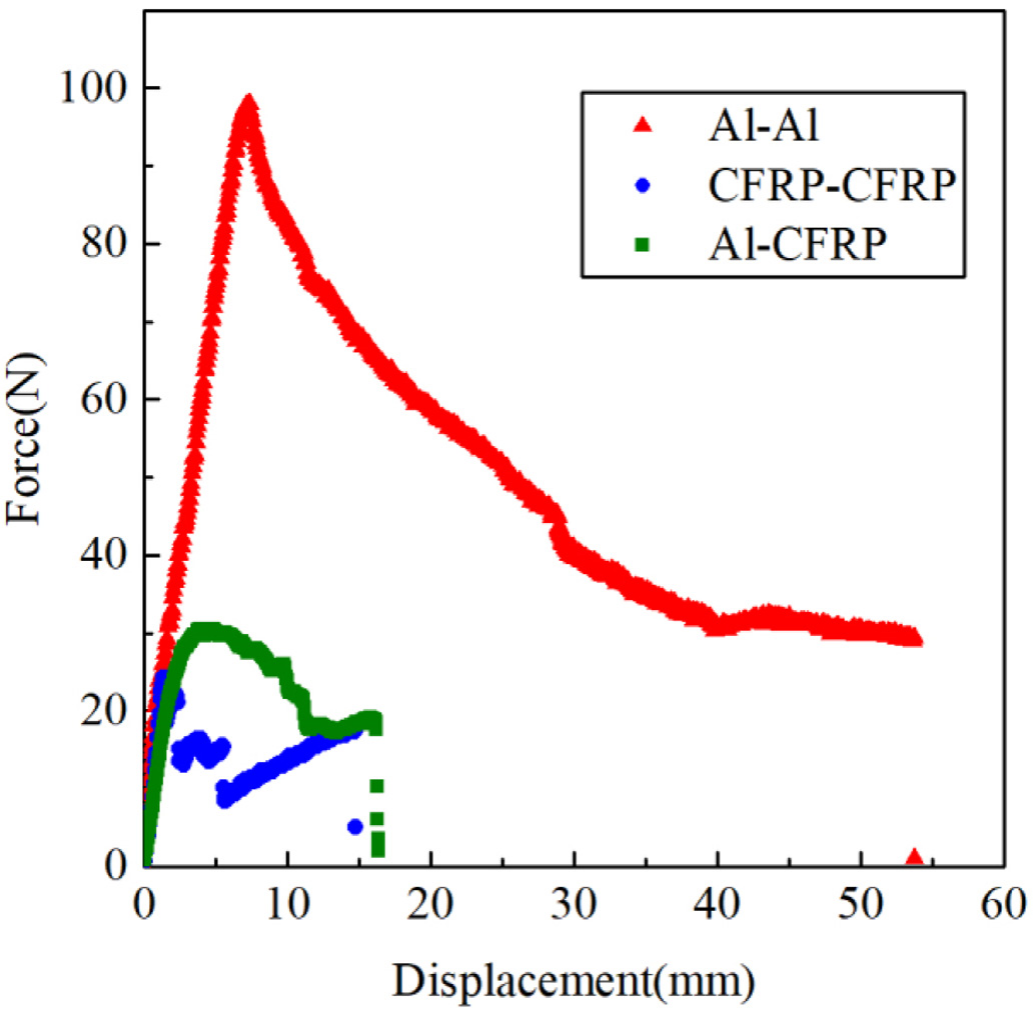
Comparison of loading curves of different specimens.

Cracks in Al–Al and CFRP–CFRP specimens grew steadily, and the cracks in the CFRP-aluminum specimen piece exhibited unstable growth. The load-displacement curves were recorded in the test, which is shown in Figure 3.

The ultimate load of different specimens is listed in Table 2.

**Table 2:** Ultimate load of different specimens.

Specimens type	Ultimate load(N)
CFRP–CFRP	30.59
Al–Al	97.96
Al-CFRP	24.35

It can be found from Figure 3 and Table 2 that the load-displacement curves of the three different specimens are quite different. Before the peak load, the load-displacement curves of the three test pieces all change approximately linearly. The peak load of the Al–Al test piece is the largest, the peak load of the CFRP–CFRP test piece is close to that of the Al-CFRP test piece, and the peak load of the CFRP–CFRP test piece is slightly higher. The loading curves of the three test pieces at the softening stage after the peak load show completely different laws. The loading curve of the Al–Al test piece is relatively smooth, and there is no obvious jump. The loading curve of the Al-CFRP test piece has an obvious sudden drop, and the loading curve of the CFRP–CFRP test piece also has a sudden change, but it is not as obvious as that of Al-CFRP.

**Figure 4:**
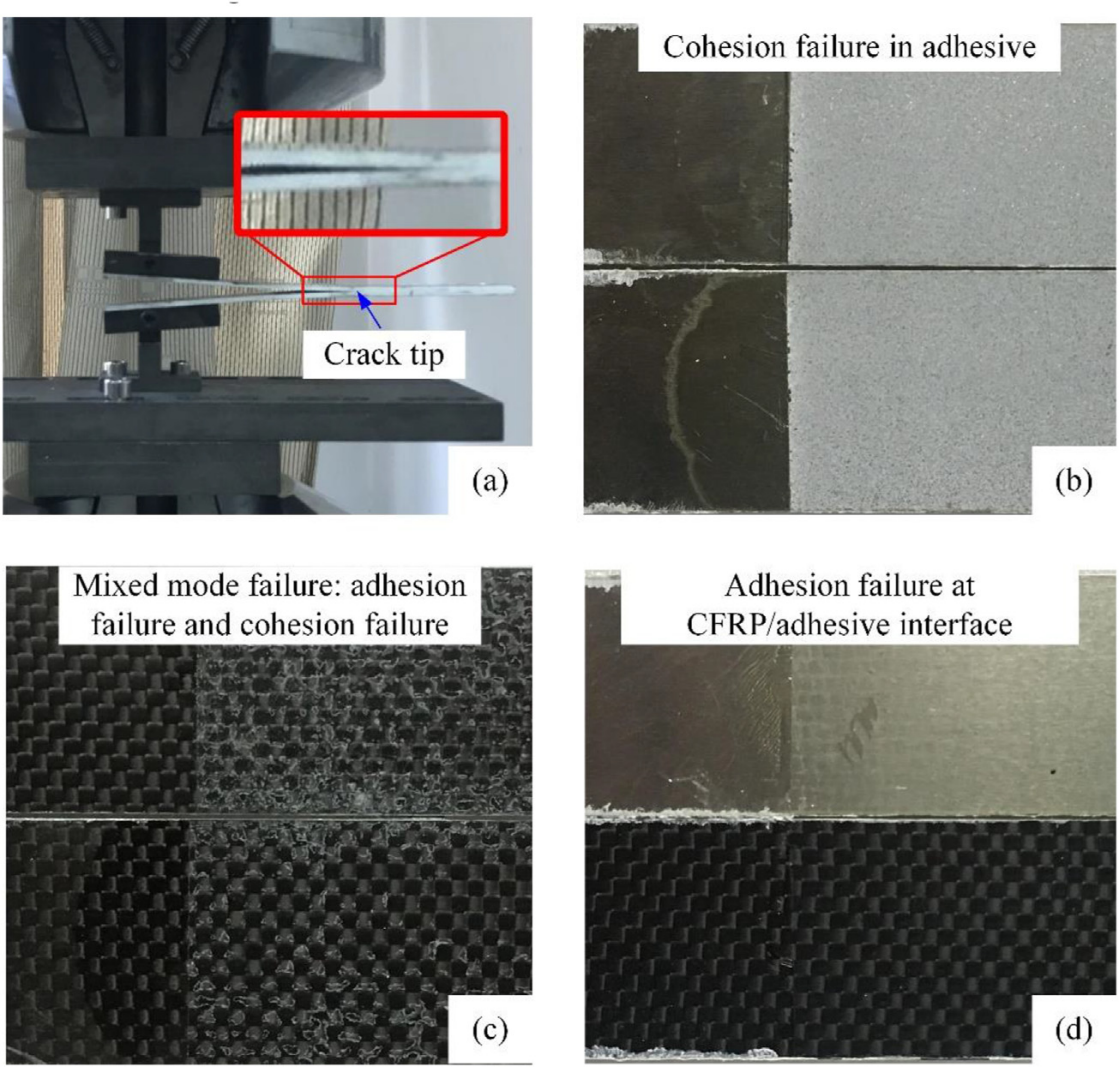
Failure modes of different bonding test pieces.

After the test piece is completely separated, the failure mode of the bonding surface is shown in Figure 4.

**Figure 5:**
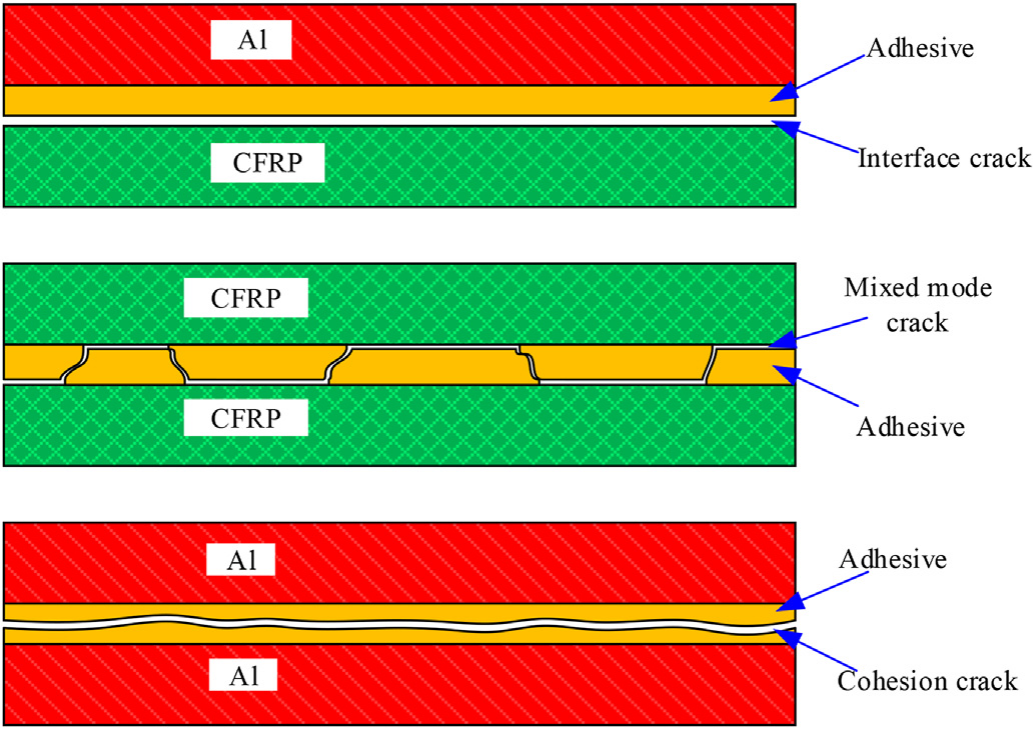
Comparisons of failure modes of different test pieces.

It can be seen from Figure 4 that the three different types of specimens exhibit completely different failure modes. Figure 5 compares the differences between the three types of test pieces.

The failure mode of the Al–Al specimen is entirely the cohesive failure in the adhesive layer, and no interface failure between the adhesive layer and the Al plate occurred during the crack propagation process. The failure mode of the Al-CFRP specimen is completely the interface failure between the adhesive layer and the CFRP board. The cohesive failure of the adhesive layer and the interface failure were both observed in CFRP–CFRP specimen and cracks migrates between adhesive and interface.

## 3 Critical energy release rate

The critical energy release rate is an important indicator for characterizing the bonding performance

(1) $$G_{C}=\frac{\Delta U}{B\Delta a}$$

The calculation method of the critical energy release rate of mode I crack in the asymmetric DCB test is [29].

(2) $$G_{\text{IC}}=\frac{P^{2}a^{2}}{2b}\left(\frac{1}{E_{1}I_{1}}+\frac{1}{E_{2}I_{2}}\right)$$

where *P* is the critical load, *a* is the crack length corresponding to the critical load, *b* is the width of the test piece, and *E*_1_*I*_1_ and *E*_2_*I*_2_ are the bending stiffness.

Three methods of defining the critical load are recommended in the ASTM D5528, including *P*_vis_, *P*_max_ and *P*_5%_. In this paper, *P*_max_ is used as the critical load for crack growth. The critical crack length corresponding to the critical load in the DCB test is taken as 55 mm.

According to Eq. (2), the critical energy release rates of three different test pieces can be obtained and listed in Table 3.

**Table 3:** Mode I CERR of different test pieces.

Specimen	*G*_IC_ (J/m^2^)
Al-CFRP	42.93
Al–Al	694.88
CFRP–CFRP	67.76

It can be seen from Table 3 that the failure mode of the bonding interface has a significant impact on the critical energy release rate. The failure mode of the Al–Al specimen is completely the cohesive failure of the adhesive layer and has the highest critical energy release rate. Both the Al-CFRP test piece and the CFRP–CFRP test piece have the interface failure between the adhesive layer and the board, and the critical energy release rate is much lower than that of the cohesive failure. At the same time, it can be found that the critical energy release rate of CFRP–CFRP is higher than that of Al-CFRP. According to Figure 4, cohesive failure occurred in the adhesive layer during the failure of the CFRP–CFRP specimen, and more energy can be absorbed due to the adhesive failure. According to Eq. (1), the damage in the adhesive layer can lead to a higher critical energy release rate in the CFRP–CFRP test piece than that in the Al-CFRP test piece.

## 4 Finite element simulation

### 4.1 FEM methods

At present, many finite element methods have been used to study the fracture behavior of the adhesive layer. It mainly includes the cohesive zone model, the virtual crack closure technology and the extended finite element. Rarani [18] simulated the delamination behavior of the DCB test through these methods, and discussed their advantages and disadvantages.

The cohesive zone model defines the relationship between the traction in the cohesion zone and the opening displacement of the interface. The constitutive law can be described by Eq. (3)

(3) $$\left\{\begin{array}{c} t_{n}\\ t_{s}\\ t_{t} \end{array}\right\}=\left[\begin{array}{ccc} K_{n} & 0 & 0\\ 0 & K_{s} & 0\\ 0 & 0 & K_{t} \end{array}\right]\left\{\begin{array}{c} \delta_{n}\\ \delta_{s}\\ \delta_{t} \end{array}\right\}$$

where, *t*_*i*_ is the traction force, *K*_*i*_ is the interface stiffness, *δ*_*i*_ is the opening displacement, *i* = n, s, t.

After the crack initiation, softening occurs in the material. The softening process is described by introducing the damage variable *D*, as shown in Eq. (4)

(4) $$\begin{array}{c} t_{n}=\left\{ \begin{array}{cc} \left(1-D\right){\bar{t}}_{n} & {\bar{t}}_{n}\geq 0\\ {\bar{t}}_{n} & {\bar{t}}_{n}<0\; \end{array}\right.\\ t_{s}=\left(1-D\right){\bar{t}}_{s}\\ t_{t}=\left(1-D\right){\bar{t}}_{t} \end{array}$$

where, ${\bar{t}}_{n},{\bar{t}}_{s},{\bar{t}}_{t}$ are the stresses predicted by the elastic traction-separation behavior for the current strains without damage.

**Figure 6:**
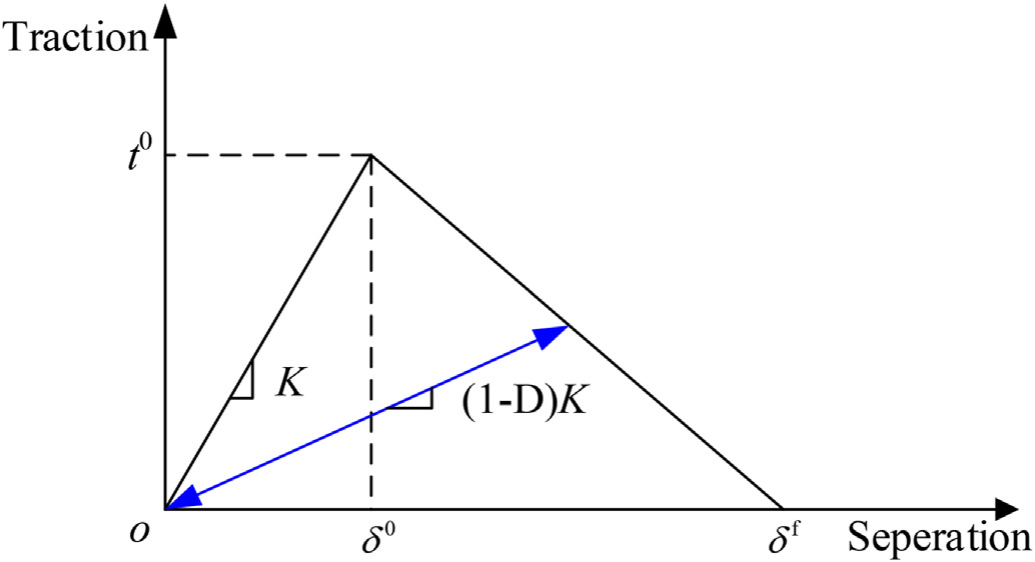
Bilinear cohesive model.

The typical traction-separation response of bilinear cohesive model is shown in Figure 6.

There are four main crack initiation criteria: maximum nominal stress criterion, maximum nominal strain criterion, quadratic nominal stress criterion and quadratic nominal strain criterion. Damage is assumed to initiate when the initiation criterion reaches a value of one. The failure of the cohesive element is generally determined by a criterion based on the energy release rate. At present, the main failure criteria include the Reeder criterion, the BK criterion and the power law criterion [30]. In this study, the crack is assumed to be pure mode I. The maximum nominal stress initiation criterion and power law failure criterion are adopted.

**Figure 7:**
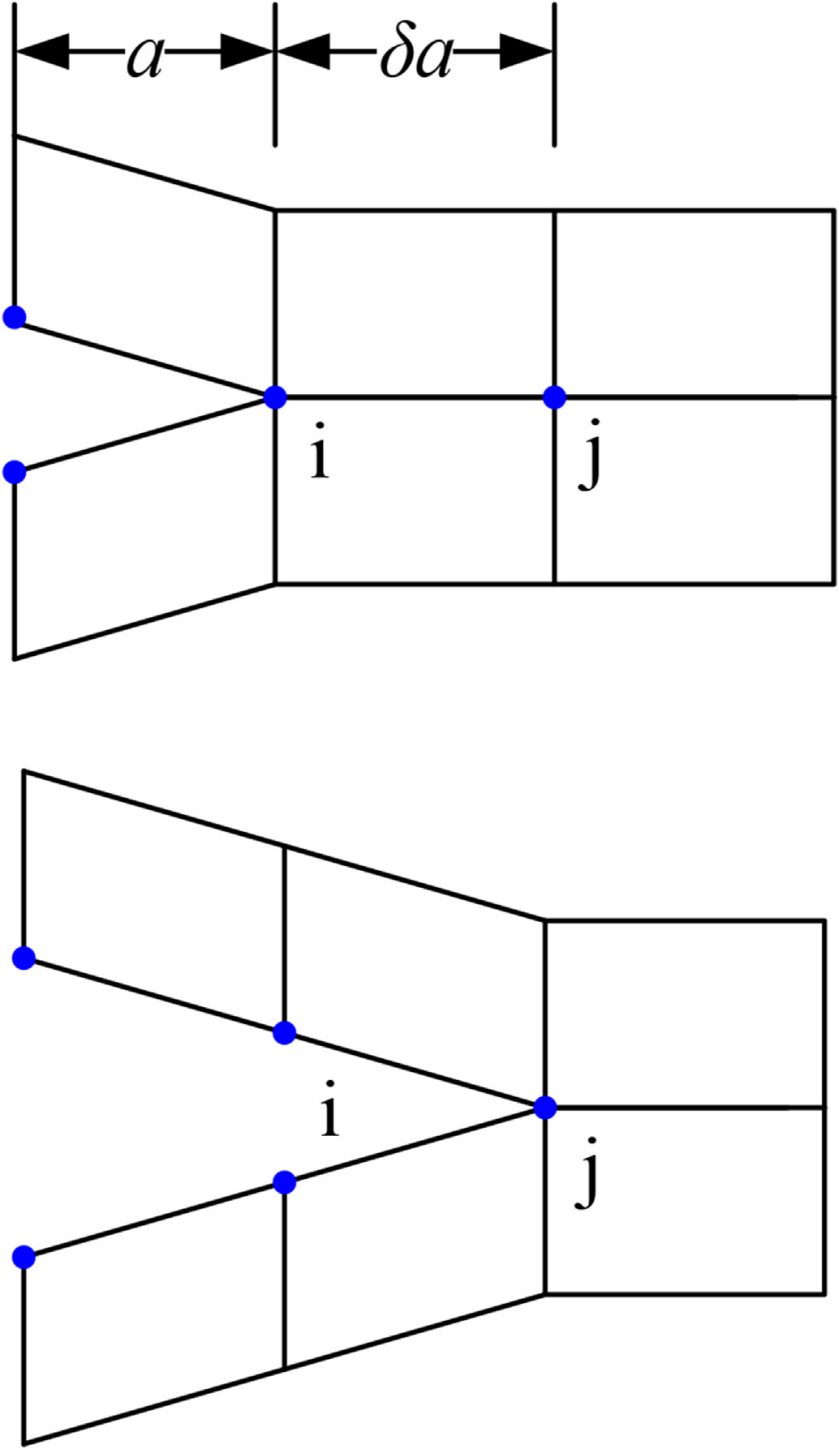
Schematic of VCCT

Irwin [31] proposed the VCCT technology where the energy consumed by opening the crack is believed to be equal to the energy required to close the crack in the process of crack propagation, as shown in Figure 7.

The mode I crack energy release rate can be expressed by Eq. (5)

(5) $$G_{I}=\frac{Fv}{2b\delta a}$$

where, *F* is the force on the node, *v* is the relative displacement of the node, and *b* is the width.

When the energy release rate meets Eq. (6)

(6) $$f=\frac{G_{I}}{G_{IC}}>1$$

the constraints between the nodes are released and the cracks propagate.

The XFEM is an extension of traditional finite element methods. Belytschko [32] proposed an extended finite element method. The traditional finite element method has the following problems when analyzing the fracture problem: 1) The crack tip mesh needs to be refined to accurately calculate the crack tip stress field and stress intensity factor; 2) The crack propagation process needs to be re-meshed. Compared with the traditional finite element method, the cracks in XFEM can grow inside the element, there is no need to re-mesh during the crack propagation, and the crack tip does not need to be densified.

**Figure 8:**
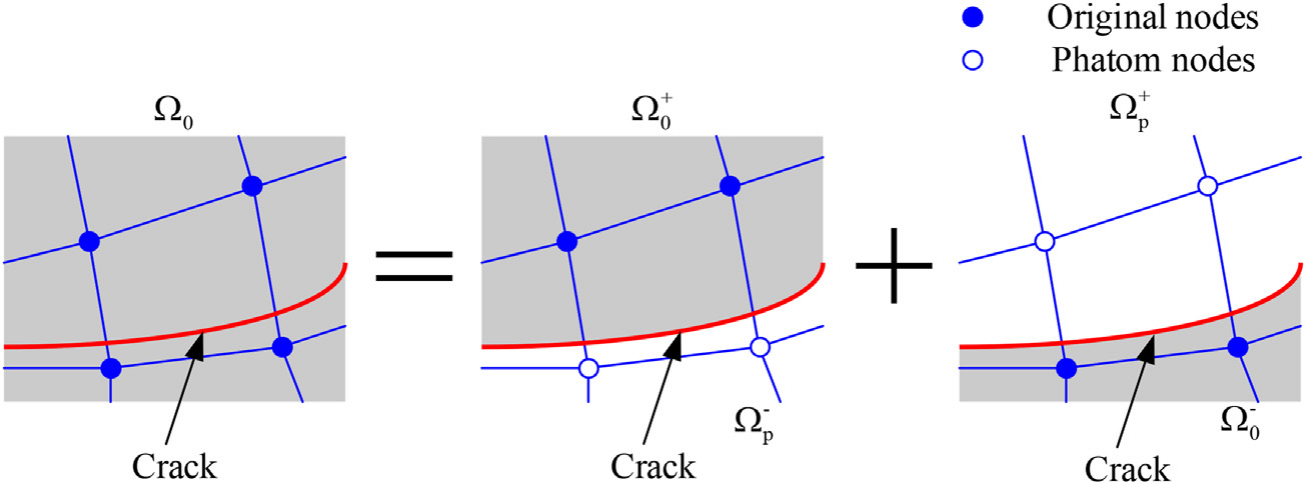
Schematic of XFEM

As shown in Figure 8, when the element is intact, the virtual node is completely constrained to the corresponding initial node. When the element is penetrated by the crack, the element is split into two parts by the crack, and the constraint between the virtual node and the initial node no longer works.

The displacement of any Gauss point in the element of the extended finite element is described Eq. (7)

(7) $$u=\sum_{I=1}^{N}N_{I}\left(x\right)\left[u_{I}+H\left(x\right)a_{I}+\sum_{\alpha =1}^{4}F_{\alpha }\left(x\right)b_{I}^{\alpha }\right]$$

where, *F* is the crack tip enhancement function, *H*(*x*) is the jump function at Gauss point *x*, on one side of the crack surface, *H* = 1 and on another side, *H* = −1. *u*_*I*_ is the displacement vector, *a*_*I*_ generates the vector of the enriched degree of freedom, *bα I* is the enriched nodes degree of freedom vector.

### 4.2 Finite element model

Three failure modes are observed in the experiment: the cohesive failure of the adhesive, the interface failure between the adhesive and the board, and the mixed failure in the adhesive and interface. The CZM, VCCT, and XFEM methods are adopted to numerically simulate the DCB test. The bilinear constitutive is used in the CZM model, and the interfacial stiffness and strength are 10^5^ N/mm and 20 MPa [27, 28], respectively. Thickness of adhesive is assumed to be 0.1mm in XFEM model.

**Figure 9:**
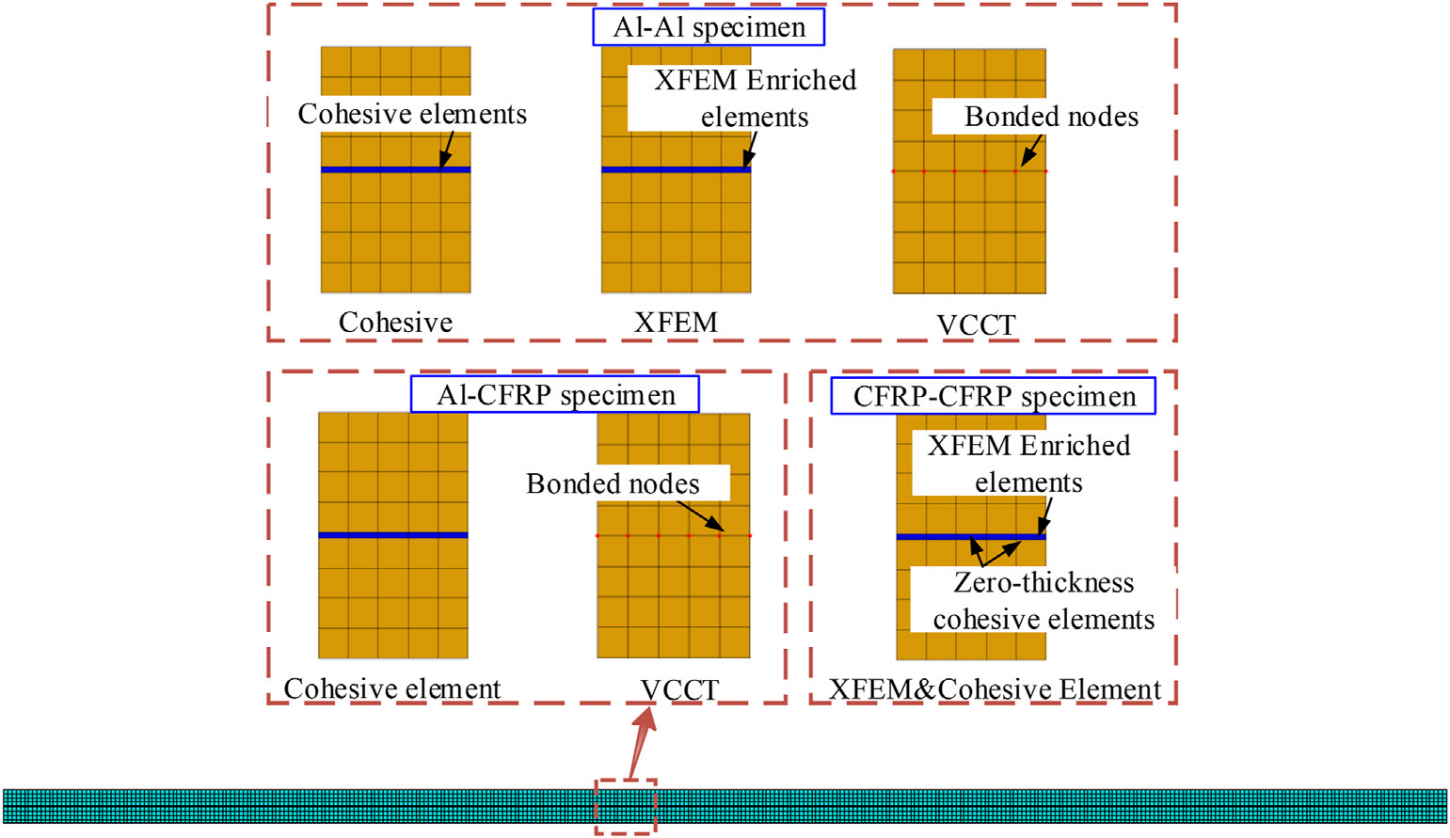
FEM modeling methods of different specimens.

The finite element (FE) models are shown in Figure 9. FE models are established with Abaqus/Standard. 2D plane strain elements (CPE4R) are applied for the beam and cohesive elements (COH2D4) are applied for the adhesive. A mesh sensitivity analysis has been carried out in the following sections, and the size of elements are chosen as 0.1 mm.

According to the test results, when interface failure occurs, the crack propagation shows an obvious randomness. The randomness of interface performance is introduced into the finite element model to account for the influence of randomness. This paper assumes that the interface performance distribution satisfies the Gaussian distribution in Eq. (8)

(8) $$X∼N\left(\mu ,\sigma^{2}\right)$$

where, *X* is a random variable, *μ* is the mean value, and *σ* is the variance.

Through the Box-Muller transform [33], the random material properties that obey the Gaussian distribution are obtained, and the randomness is introduced into the finite element model through the UFIELD (VCCT) and USDFLD (CZM) subroutines.

### 4.3 Results and discussion

**Figure 10:**
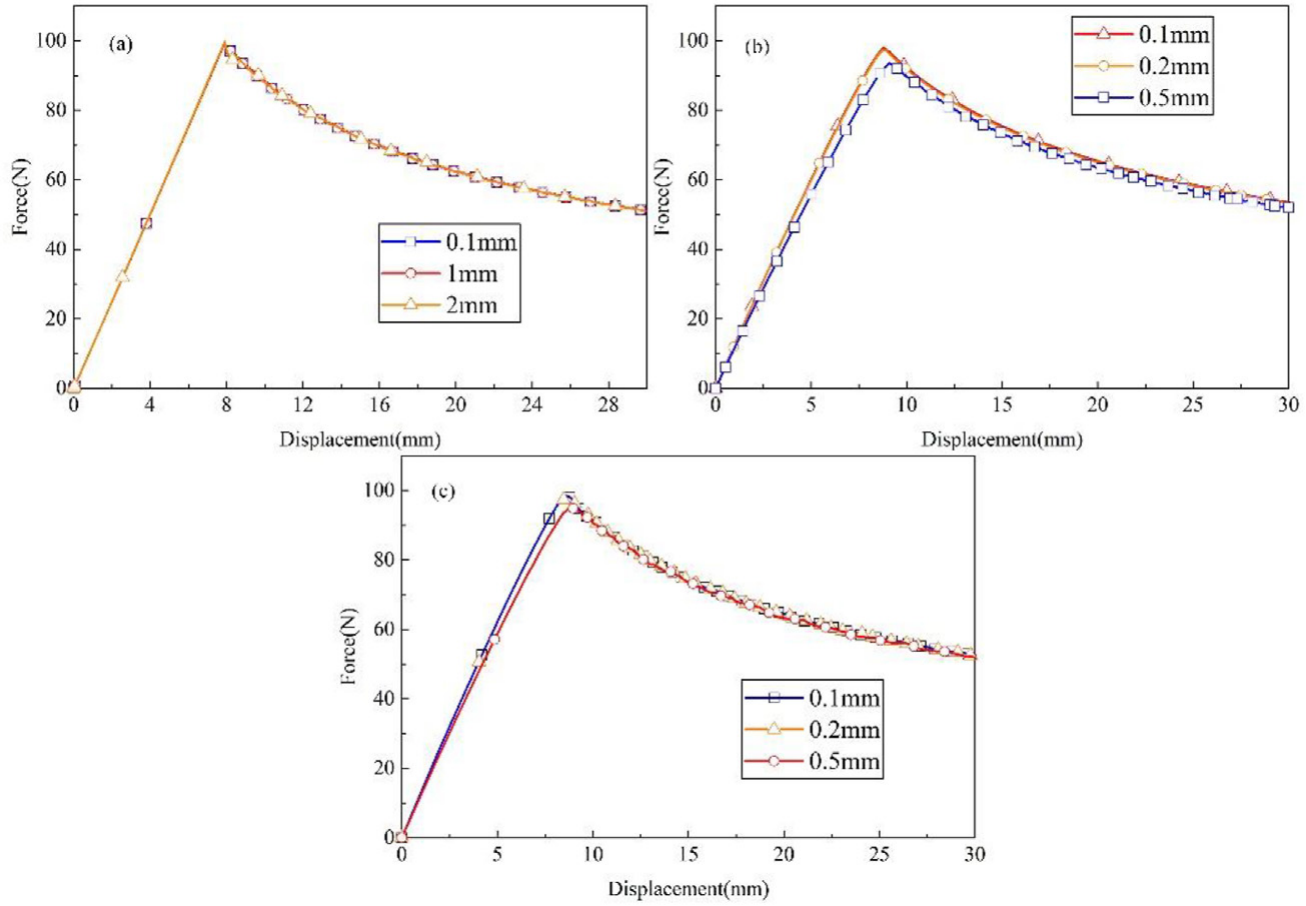
Mesh sensitivity analysis of different modeling strategies (a) VCCT (b) CZM (c) XFEM.

A mesh sensitivity analysis has been carried out to ensure the convergence of element size. The VCCT model is meshed with 0.1 mm, 1 mm and 2 mm size elements. CZM and XFEM model is meshed with 0.1 mm, 0.2 mm and 0.5 mm size elements. The comparisons of load-displacement curves are shown in Figure 10. The VCCT model shows nearly no sensitivity to the mesh size, and the results converged when the size is smaller than 2 mm. Element sizes influence the initial stiffness and peak load of CZM and XFEM model. The initial stiffness and peak load increases with the decreasing of element size. 0.1 mm is small enough to obtain an ideal result for XFEM and CZM model.

**Figure 11:**
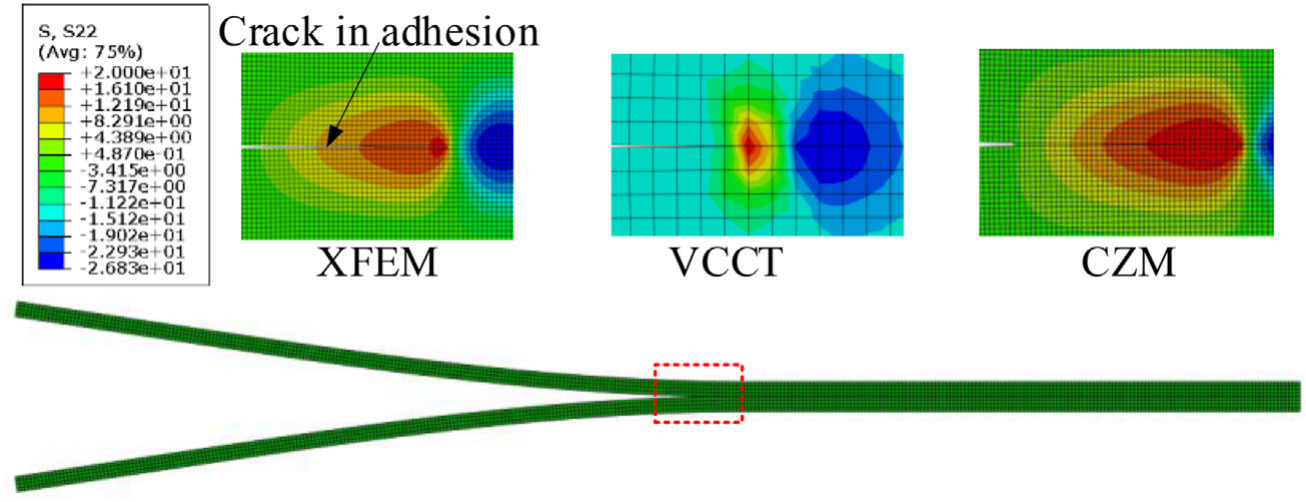
Comparison of stress distribution of XFEM, VCCT and CZM.

Figure 11 compares the stress field distribution at the crack tip of Al–Al specimen with different numerical models. It can be found that there exists a spindle-shaped cohesion influence zone at the crack tip of XFEM and CZM, which is obviously different from the results of VCCT.

**Figure 12:**
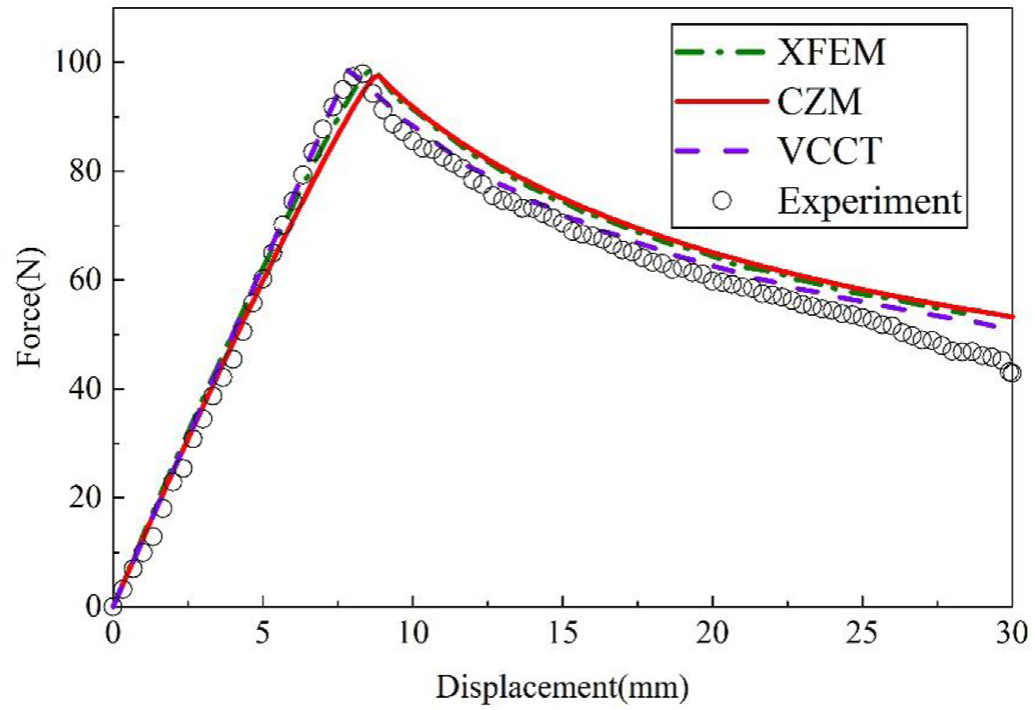
Loading curves of different FE methods for Al–Al specimen.

Figure 12 shows the comparison of results between numerical models and the experimental data. The peak loads calculated by CZM, XFEM and VCCT are respectively 98.05 N, 98.51 N and 99.20 N, which are close to the experimental result of 97.94 N. At the same time, it can be found from Figure 12 that the results of VCCT at the softening stage are closer to the test results, while the results calculated by XFEM and CZM are larger.

**Figure 13:**
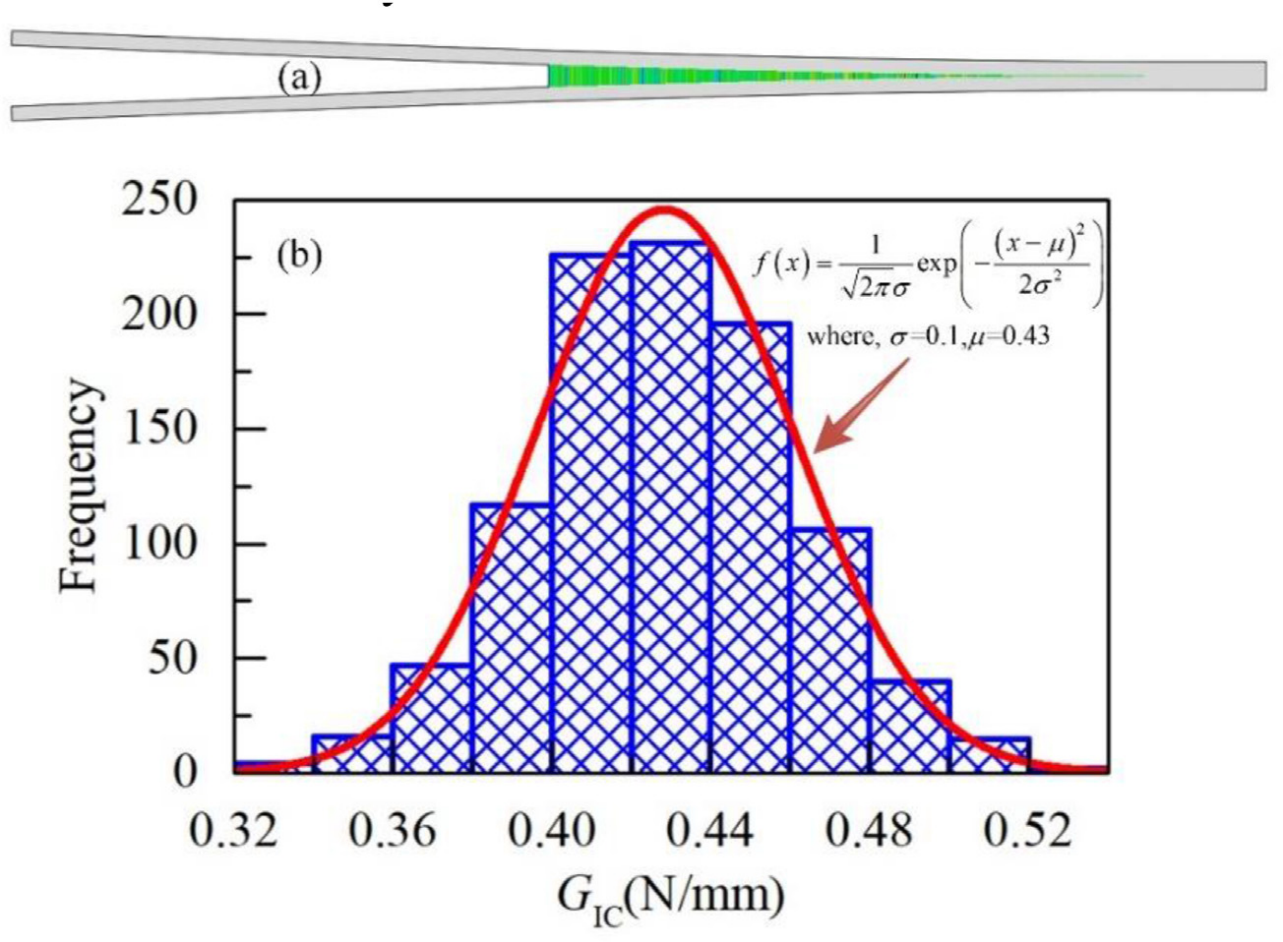
Gaussian distribution of material properties in simulation.

The material randomness is introduced to the interface of Al-CFRP and CFRP–CFRP specimen, as shown in Figure 13. Figure 13a is the material property cloud diagram of the interface, and Figure 13b is the statistical data of the material properties. It can be found that the randomness of materials introduced by the Box-Muller transformation obeys the Gauss distribution.

**Figure 14:**
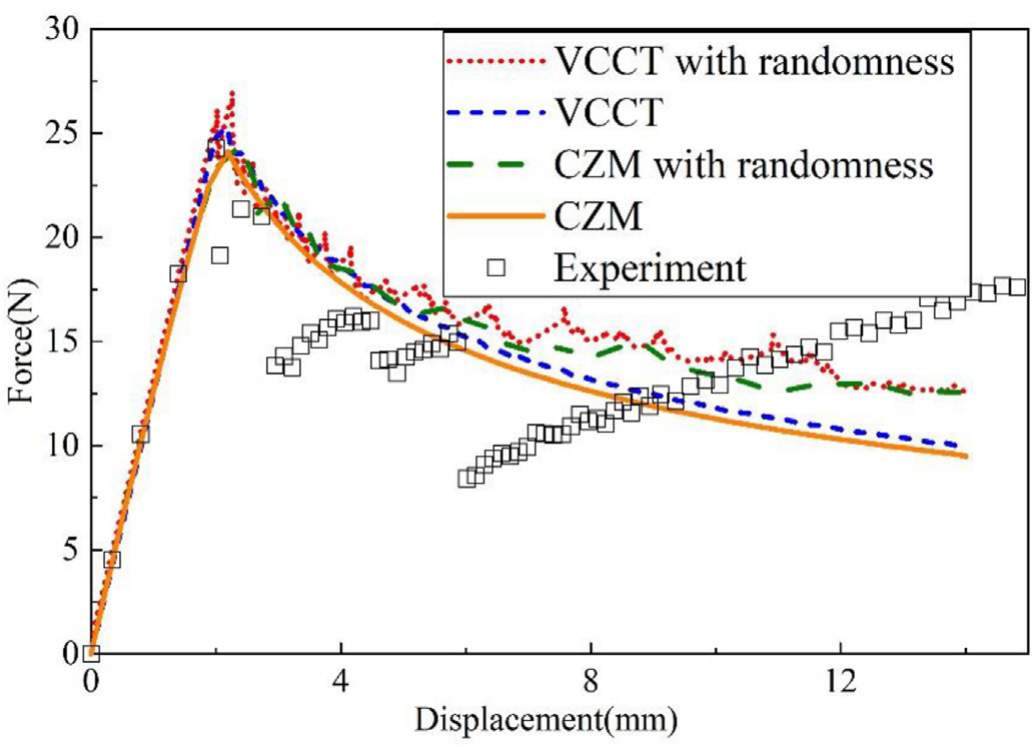
Influence of randomness on loading curves for Al-CFRP specimen.

Figure 14 compares the effect of material randomness on the simulation results. It can be found that when the material randomness is not introduced, the results calculated by VCCT and CZM are smoother at the descending stage, which is quite different from the experimental results. After introducing the randomness of the material, the calculated result shows a jagged shape similar to the test result. When the crack propagates to the element with lower fracture toughness, the load will drop suddenly, and the element with better performance will cause the load to increase, which leads to the irregularity and discontinuities of the predicted load-displacement curve.

**Figure 15:**
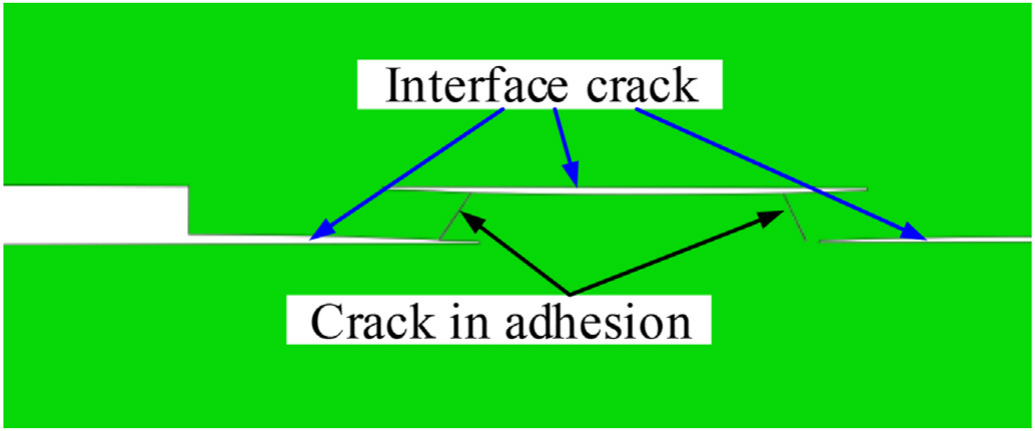
Interface failure and cohesive failure for CFRP–CFRP specimen.

Failure mode of CFRP–CFRP specimen is shown in Figure 15. Cracks in the interface migrates through the adhesive to the other side, which is consistent with the test results shown in Figure 5. From the simulation results, it can be known that due to the dispersity between the interfaces, discontinuous delamination will appear on both sides of the adhesive layer, which causes the adhesive to bend during the loading process, and thereby generates additional bending stress. Therefore, although the strength of the adhesive layer is higher than the strength of the interface, the additional bending stress on the adhesive layer can still cause the adhesive layer to break.

**Figure 16:**
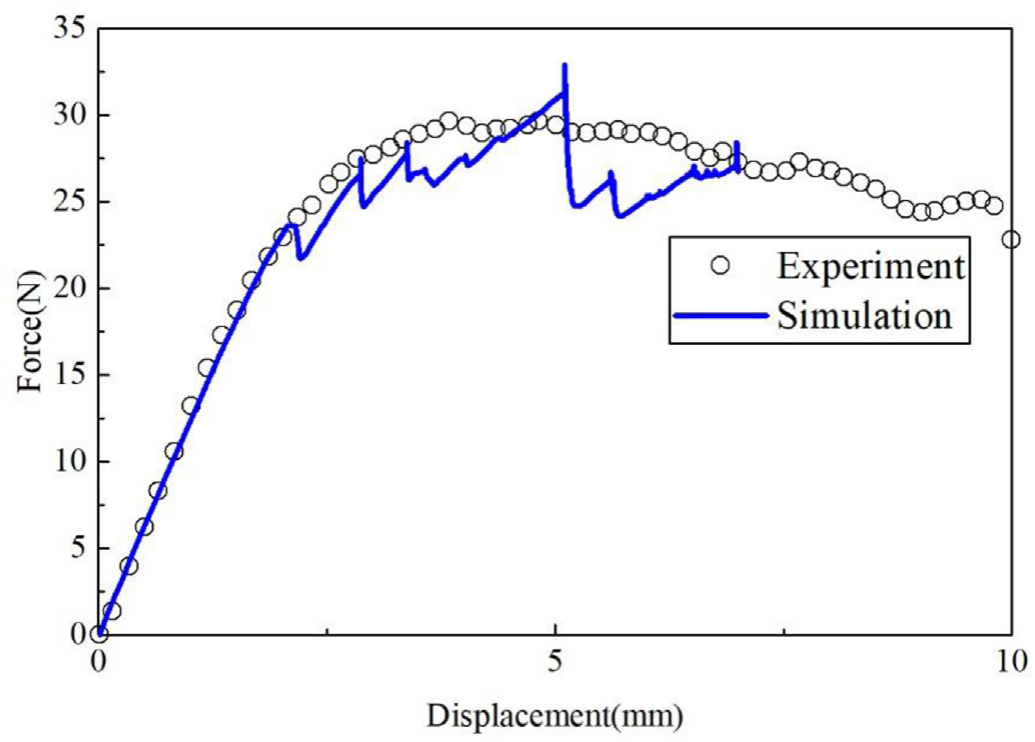
Load-displacement curve of CFRP–CFRP specimen.

Figure 16 compares the load-displacement curves of the simulation and the experiment. The predicted result shows a good agreement with the experimental result. The delamination initiation of CFRP–CFRP specimen followed by a stable softening shows a great difference from the Al-CFRP specimen in Figure 14. Cracks migrate in the adhesive and dissipate more energy and the bridging effect of the adhesive improves the strength and fracture toughness compared to Al-CFRP specimen.

## 5 Conclusion

A series of DCB tests have been carried out to investigate the bonding behavior between different materials. Three different numerical strategies are adopted to predict the failure behavior of different specimens. A XFEM/CZM coupled approach was proposed to predict the crack migration phenomena in CFRP–CFRP specimen. A Weibull distribution of the interfacial property is introduced to investigate the unstable propagation of cracks in Al-CFRP specimen. The predicted results are in close agreement with the experiment. The following conclusions can be obtained.

1. The base material has a significant effect on the bonding performance. In this study, failure mode of Al–Al specimen is cohesive destruction and which lead to the best bonding performance.
2. Randomness of the interface will lead to the unstable propagation of cracks and the bonding strength decreases obviously for interface failure. It is of great significance to introduce the randomness in simulating interface failure.
3. Crack migrations in the adhesive dissipate more energy and slightly increase the bonding performance compared to pure interface failure. The XFEM/CZM coupled model can effect predict the failure process of CFRP–CFRP specimen.

## Declarations

### Author contribution statement

Mamingze: Conceived and designed the experiments; Performed the experiments; Analyzed and interpreted the data; Contributed reagents, materials, analysis tools or data; Wrote the paper.

Zhiqiang Zhou: Analyzed and interpreted the data; Contributed reagents, materials, analysis tools or data; Wrote the paper.

Jiang Wen: Performed the experiments.

Yao Weixing: Contributed reagents, materials, analysis tools or data.

Li Piao: Analyzed and interpreted the data; Wrote the paper.

### Funding statement

Weixing Yao was supported by 10.13039/501100001809National Natural Science Foundation of China (52075244).

### Data availability statement

Data will be made available on request.

### Declaration of interest’s statement

The authors declare no conflict of interest.

### Additional information

No additional information is available for this paper.
